# Exploring Culinary Tourism and Female Consumer Preferences for Selected National Cuisines in Poland: A Sensory and Preference Analysis of Food Products from Four Countries

**DOI:** 10.3390/foods14010073

**Published:** 2024-12-30

**Authors:** Agata Kiciak, Wiktoria Staśkiewicz-Bartecka, Natalia Kuczka, Agnieszka Bielaszka, Marzena Tudrej, Oskar Kowalski, Marek Kardas

**Affiliations:** 1 Department of Food Technology and Quality Assessment, School of Public Health in Bytom, Medical University of Silesia in Katowice, ul. Jordana 19, 41-808 Zabrze, Poland; wstaskiewicz@sum.edu.pl (W.S.-B.); natalia.k4940@gmail.com (N.K.); abielaszka@sum.edu.pl (A.B.); mkardas@sum.edu.pl (M.K.); 2 Department Human Nutrition, Department of Dietetics, Faculty of Public Health in Bytom, Medical University of Silesia in Katowice, ul. Jordana 19, 41-808 Zabrze, Poland; okowalski@sum.edu.pl

**Keywords:** world cuisines, consumer preferences, sensory analysis, culinary tourism

## Abstract

Background/Objectives: The development of culinary tourism offers not only unique culinary travel experiences but also allows for the exploration of various aspects related to food. The main aim of this study was to assess the food preferences of a selected group of female consumers regarding world cuisine and to analyze the sensory quality of selected world cuisine products: ayran, rice noodles, tempeh, and chorizo. Methods: Sensory evaluation of utility characteristics, including color, aroma, texture, appearance, and taste, was conducted using a five-point scale. A custom questionnaire was used to collect data on respondents’ preferences and demographic characteristics. This study included 51 sensory panelists and 356 survey participants. Results: Among the evaluated products, rice noodles received the highest median rating (Me = 4.8), while tempeh scored the lowest (Me = 3.8). Statistical analysis revealed significant differences in sensory perceptions depending on prior product familiarity. Italian (67.5%) and Polish (65.8%) cuisines were most frequently preferred, whereas Indian cuisine (4.3%) was the least popular. Additionally, over 83% of respondents indicated they regularly patronize food establishments offering regional dishes during travel. Conclusion: This study highlights a strong preference for familiar cuisines, such as Italian and Polish, among Polish female consumers, with implications for targeted marketing strategies in the gastronomy sector. The sensory analysis provides actionable insights into product acceptance, emphasizing the importance of cultural adaptation in promoting international food products.

## 1. Introduction

In recent years, culinary tourism has gained significant attention from scholars and practitioners due to its central role in travel experiences and its influence on the behavioral intentions of tourists. This phenomenon is particularly relevant in the context of globalization and the growing demand for authentic culinary experiences [1,2,3]. The early 21st century marked a notable rise in Poles’ interest in nutrition, meal preparation, and discovering new, unfamiliar flavors through travel [4]. In this context, food serves as both an essential cultural element and a reflection of consumer dietary preferences, which are shaped by cultural background, product availability, and global health trends [5,6].

Despite growing interest, previous studies on consumer preferences for world cuisines have largely relied on generalized surveys, often overlooking detailed behavioral and cultural nuances [7]. Furthermore, many such studies were conducted on foreign populations, failing to address the unique characteristics of Polish consumers [8]. This study addresses these gaps by employing a five-point sensory evaluation scale and considering respondents’ familiarity with the tested products. These methodological advancements provide a more nuanced and precise understanding of Polish consumers’ preferences, contributing significantly to the existing literature.

Food consumption satisfies not only physiological needs but also fulfills critical social and psychological roles. Beyond providing essential nutrients, food fosters social bonds and reinforces cultural identity. Consequently, food plays a pivotal role in creating and sustaining cultural connections across communities and nations [5]. Culinary tourism, with its dual impact on gastronomy and tourism, has emerged as a vital aspect of cultural exchange, as tourists often associate their travel destinations with the local foods they experience [9].

This study aligns with broader global socio-economic trends. Globalization has expanded access to diverse culinary products, enhancing consumer choice while presenting challenges for local market adaptation [10,11]. Concurrently, rising health awareness among consumers has shifted dietary preferences toward natural and minimally processed foods. These evolving attitudes influence interest in specific cuisines, a trend that this study seeks to explore within the Polish context [12].

Culinary tourism is currently one of the fastest-growing segments of cultural tourism [13]. Its appeal extends beyond food consumption to encompass unique experiences, such as participating in cooking workshops, interacting with local chefs, and exploring regional culinary traditions [4,9]. Traditional regional cuisine has become an integral part of leisure activities, even when gastronomy is not the primary purpose of travel. Notably, dining and sampling local products are often regarded as some of the most enjoyable travel activities, with minimal financial constraints [4,9].

Socio-cultural trends and population migrations continue to reshape culinary traditions and eating habits. Younger generations, in particular, frequently adapt their culinary preferences through international exposure and travel experiences [14]. Technological advancements, especially in social media, have further transformed tourism by enabling consumers to create, share, and access culinary experiences on a global scale [15].

A review of the literature reveals a lack of critical analysis of theoretical frameworks in prior studies. Many frameworks neglect the effects of globalization and health trends on consumer behavior and fail to consider local market dynamics [16,17,18]. This study adopts a comprehensive approach, integrating sensory and behavioral aspects to capture not only taste preferences but also the broader socio-cultural contexts influencing consumer choices. Such an approach facilitates a deeper understanding of Polish consumers’ reactions to international cuisine products.

Culinary traditions and dietary habits are in constant flux, shaped by migration, cultural shifts, and travel experiences. These changes are particularly evident among younger populations [14]. Additionally, technological progress has revolutionized the way tourists access and share information, making social media a powerful tool for influencing tourism trends [15]. Content shared online now plays a critical role in shaping perceptions and outcomes in the tourism industry.

With evolving social structures, the role of gastronomy has expanded beyond satisfying hunger. Gastronomic establishments now offer multifaceted experiences, fulfilling social, cultural, and psychological needs. These dynamics apply not only to local consumers but also to tourists seeking unique culinary encounters [19,20].

Local dishes, as intangible cultural heritage, provide tourists with authentic cultural experiences. Some culinary traditions date back centuries, preserving cultural identity while serving as a powerful tool for promoting culinary tourism [9]. Tourism itself serves as a gateway to exploring new cultures and gaining diverse experiences [21]. The increasing popularity of tourism and migration has fueled interest in regional cuisines, preparation methods, and dining traditions [22].

Tourists often choose destinations based on their interest in unfamiliar culinary customs or to revisit flavors from their past [21,22]. Gastronomic services are a cornerstone of tourism, with estimates suggesting that approximately 15% of tourists are primarily motivated by high-quality traditional or regional cuisine. Such dishes and products can serve as valuable promotional tools for countries and regions [21].

In recent years, culinary tourism, encompassing cultural, cognitive, urban, rural, family, and business travel, has become increasingly popular. Defined as “the pursuit and enjoyment of unique culinary travel experiences, both near and far” [23], it is widely promoted in countries like France, Italy, and Spain for their wine trails, cheese production, olive oil, and Iberian ham. These regions exemplify how culinary tourism highlights traditional products and differentiates cuisines through unique spices and preparation methods [24]. As civilization advances, the desire to explore unfamiliar cultures grows, with culinary tourism emerging as a modern extension of ancient practices, like religious pilgrimages [25].

This study aimed to assess consumer preferences regarding preferred world cuisines and analyze the sensory quality of selected products: ayran, rice noodles, tempeh, and chorizo.

## 2. Materials and Methods

### 2.1. Sensory Experiment Design

The research material comprised four products available on the Polish market that are characteristic of different world cuisines: ayran, rice noodles, chorizo, and tempeh. The selection of ayran, rice noodles, chorizo, and tempeh as the focus of this study was driven by their distinct cultural and culinary significance, as well as their growing presence in the Polish market. Ayran represents Middle Eastern cuisine, a category gaining popularity due to its refreshing qualities and health benefits as a fermented dairy product. Rice noodles were chosen as they exemplify Asian cuisine, which has become increasingly integrated into Polish dining habits due to its versatility and alignment with health-conscious dietary trends. Chorizo, a hallmark of Spanish cuisine, was included for its unique flavor profile and representation of European culinary traditions that resonate with Polish consumers. Finally, tempeh was selected as a representative of plant-based products and Indonesian cuisine, aligning with the rising interest in sustainable and vegetarian food options. These four products were deliberately chosen to reflect diverse culinary traditions and consumption trends, allowing for a comprehensive exploration of consumer preferences for distinct world cuisines in the Polish context.

**Figure 1:**
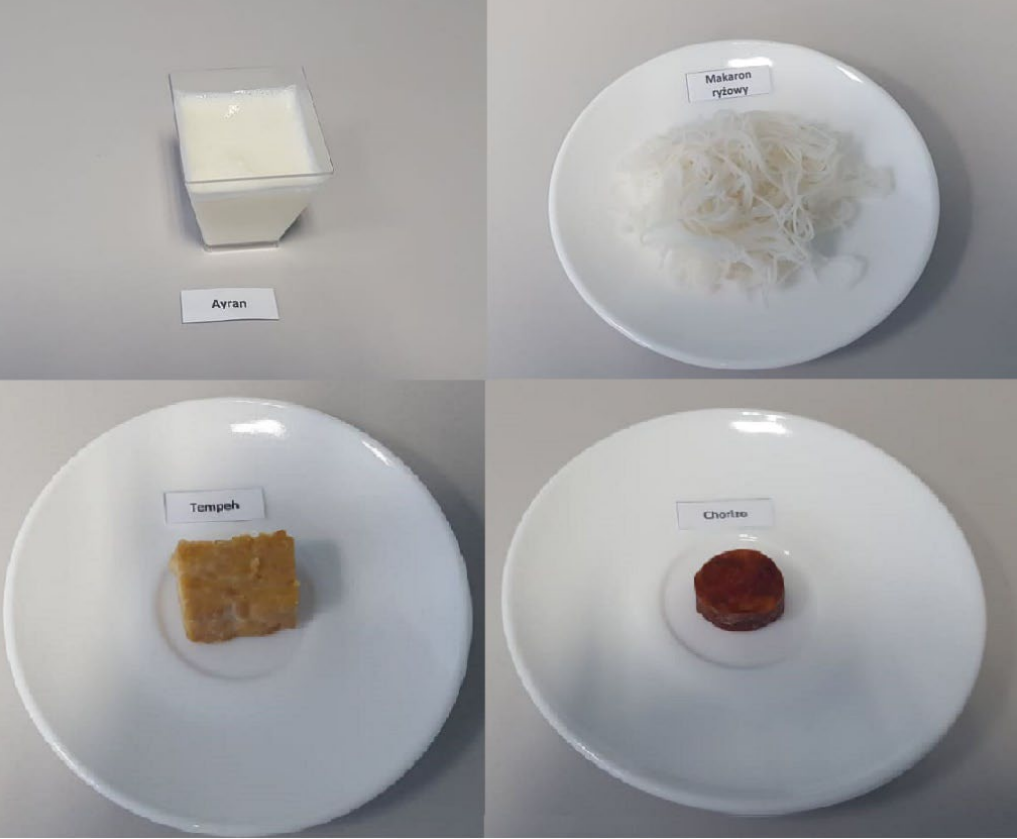
Products used for sensory quality analysis (*pl. Makaron ryżowy—ang. rice noodles*). Source: original photograph.

The selected food products prepared for this study are presented in Figure 1.

The differences between the presented products are evident and may influence how they are perceived by the evaluators. The contrast effect, resulting from distinct visual, taste, or textural features, may lead participants to focus on the most prominent aspects, which, in turn, affects their overall assessment—for example, the attractive appearance of one product may distort the perceived quality of others. Additionally, the evaluations may be prone to logical errors, such as confirmation bias, where evaluators favor samples that align with their prior experiences. In the culinary context, the diversity of food products can evoke different emotional responses, which also impacts subjective preferences (Table 1).

For this reason, it is crucial to consider these aspects when presenting samples to minimize potential evaluation errors and ensure more objective results. Understanding the cultural and emotional context in which products are consumed may contribute to a better analysis of consumer perception.

This study was conducted in the Sensory Analysis Laboratory of the Department of Dietetics at the Medical University of Silesia in Katowice, Faculty of Public Health in Bytom. The laboratory where this study was conducted met the requirements and guidelines of the PN-EN ISO 8589:2010 standard for Sensory analysis—General guidelines for the design of sensory analysis laboratories [26]. The Declaration of Helsinki of the World Medical Association guided the course of this study. The study protocol (KNW-0022/KB1/73/I/16) was reviewed and approved by the Bioethics Committee of the Medical University of Silesia in Katowice. Each participant provided informed consent for participation and was informed of the anonymity of the results.

The intensity of the assessed functional characteristics, such as color, aroma, texture, appearance, and taste, was evaluated during this study. For this purpose, a custom-designed five-point assessment sheet was used (5—very good quality of the product, 1—disqualifying quality of the product) based on international food standards contained in the Codex Alimentarius and Polish Standard PN-ISO 22935-1 for Sensory analysis of milk and milk products, Part 1: General guidance on the recruitment, selection, training, and monitoring of assessors [27].

Each participant received four coded product samples and a sheet for evaluating the individual products. Additionally, assessors received cards detailing quality attributes for all the assessed characteristics and a sheet for sensory evaluation of the samples. For each assessed attribute, a weighting coefficient was defined, by which the numerical values given by the respondents were multiplied.

This study was conducted among 51 Polish women aged 25 ± 3.05 years. The women participating in this study were qualified based on inclusion criteria that included (1) age between 18 and 30, (2) absence of a vegetarian or vegan diet, (3) no food allergies or intolerances, (4) no chronic illnesses, (5) consent to participate in this study, and (6) consent to participate in training according to PN-ISO 22935-1.

### 2.2. Consumer Preferences

The second part of this study was an assessment of consumer preferences in terms of preferred world cuisines. For this purpose, a custom questionnaire was developed, consisting of two parts. The first part included questions characterizing the respondent group in terms of gender, age, education, and place of residence. The second part of the questionnaire included questions regarding, among other things, preferred world cuisines and dining in food establishments.

The questionnaire was completed by 356 Polish women aged 23–79 years. An arbitrary sample selection was used in this study, and the questionnaire was completed by women who were shopping in one of the supermarkets in Katowice. The women participating in this part of this study were qualified based on inclusion criteria, which included (1) age over 18, (2) consent to participate in this study, and (3) complete completion of the questionnaire.

Limiting the sample selection to women only may result in findings that are difficult to generalize to the broader population. Such a selection restricts the diversity of the sample, particularly in terms of demographics, geography, and socio-economics, which may impact the full representation of Polish female consumers’ preferences for world cuisine products. Future studies could consider broader sample selection, including consumers of various genders, age groups, and regions of Poland, to obtain more representative data. Including people who shop in various stores, such as local markets, delicatessens, and supermarkets in other cities, could allow a fuller understanding of dietary preferences in different purchasing contexts.

### 2.3. Statistical Analysis

All of the obtained results were cataloged and analyzed using Microsoft 365 Excel 2022 and Statistica (v.13.3) by StatSoft Poland. The χ² test was used to analyze the relationships of non-measurable features, particularly specific questions from the second part of the survey (dietary preferences of the selected consumer group), categorized by gender and age groups (23–30 years, 31–59 years, 60–79 years). The strength of the relationship between the examined characteristics was determined using Cramér’s V coefficient, with its assessment presented based on the following scale:

- <0–0.2: weak relationship;
- <0.2–0.4: low relationship;
- <0.4–0.7: moderate relationship;
- <0.7–0.9: high relationship;
- <0.9–1: very high relationship.

The results of the intensity of the evaluated characteristics of food products were presented as mean ($\bar{x}$), standard deviation (SD), median (Me), interquartile range (Rk), minimum (xmin), and maximum (xmax). Due to the asymmetry of distributions, a non-parametric Mann–Whitney U test was used for statistical analysis between the intensity of product features and prior knowledge of the products. A significance level of *p* < 0.05 was adopted.

## 3. Results

The obtained results from the sensory evaluation using a five-point scale showed that participants rated rice noodles the highest (Me = 4.8). Among all the tested food products, tempeh received the lowest score from respondents (Me = 3.8). The results of the sensory quality assessment indicated that both taste and appearance had a significant impact on the final evaluation of each product. In particular, rice noodles received the highest ratings for taste and texture, which may be associated with their wide availability and popularity in the Polish market (Table 2).

Based on the non-parametric Mann–Whitney U test, a significantly higher taste rating for ayran was shown among individuals who were previously familiar with the tested products. For the other products (rice noodles, tempeh, and chorizo), no statistical significance was found, indicating that prior familiarity with the products did not affect the perception of the evaluated characteristics. The results of the Mann–Whitney U non-parametric test are presented in Table 3. It is worth noting that the skewness of response distributions in some cases may have influenced the test results. Outliers, particularly in the case of tempeh, may suggest significant variation in preferences, which would require further analysis considering potential cultural differences and individual preferences.

**Figure 2:**
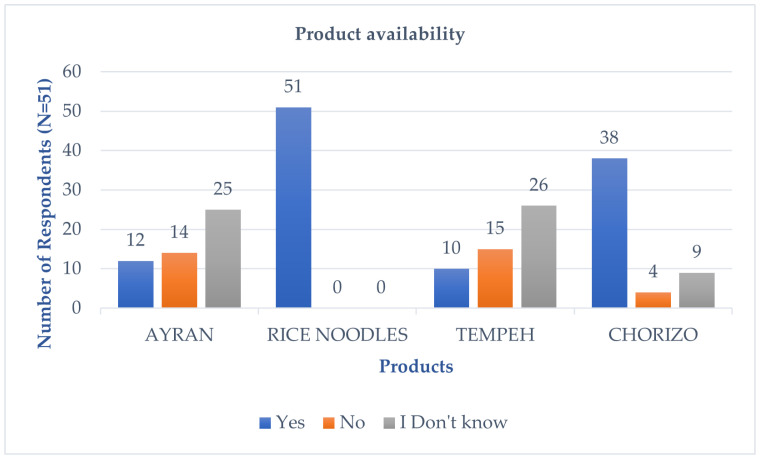
Availability of products in stores according to respondents (N = 51).

When asked about the availability of the tested products in retail, respondents indicated that rice noodles are generally available in stores (51 respondents), chorizo is available according to 38 respondents, ayran according to 12 respondents, and tempeh according to 10 respondents (Figure 2).

**Figure 3:**
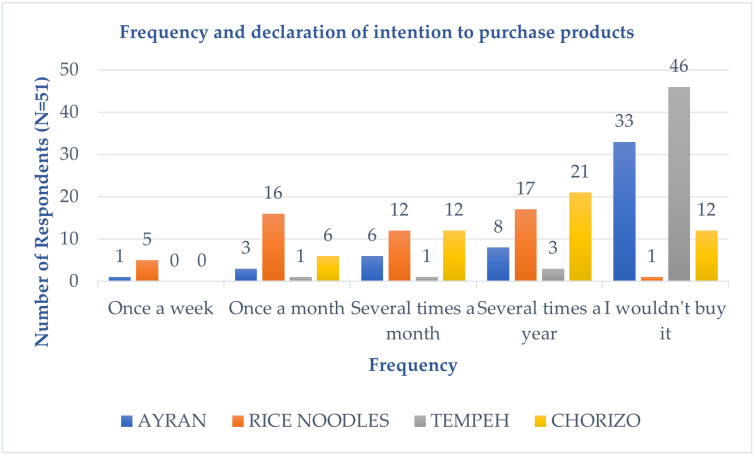
Frequency and declaration of intention to purchase products by respondents (N = 51).

In response to the question “How often would you buy the tested product?”, 46 respondents answered “I wouldn’t buy it” for tempeh, and 33 respondents for ayran. Figure 3 presents the data on the frequency and declaration of intention to purchase the products.

### 3.1. Preferred Type of World Cuisine in Dining Establishments

**Figure 4:**
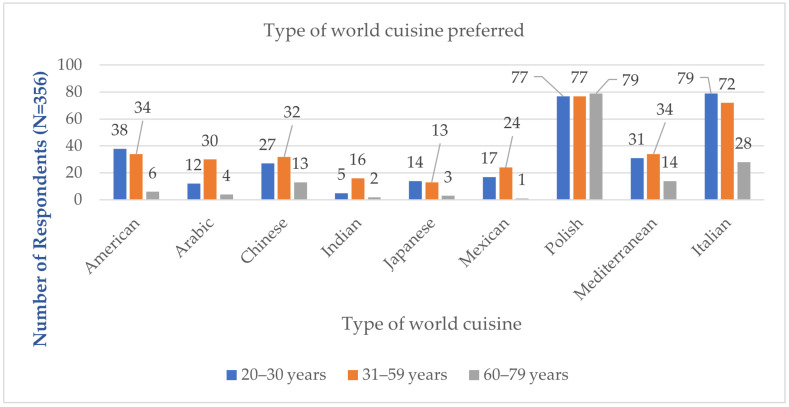
Type of preferred world cuisine (N = 356).

The most preferred world cuisine among respondents in the age group 23–30 is Italian cuisine (79 respondents) and Polish cuisine (77 respondents), while the least preferred is Indian cuisine (5 respondents). In the age groups 31–59 and 60–79, the most preferred world cuisines are Polish cuisine (77 and 79 respondents, respectively) and Italian cuisine (72 and 28 respondents, respectively). Figure 4 presents data on the type of preferred world cuisine.

Based on the χ² test, significant statistical differences were found in the preferred type of world cuisine among respondents in the age groups 23–30, 31–59, and 60–79 years. Only for the statistical feature “Polish cuisine” did the responses from participants across all age groups show no significant statistical differences. These differences may be related to cultural factors and dietary experiences shaped during different historical periods. The younger generation is more open to global influences, which explains the popularity of Italian cuisine, while older individuals are more attached to the traditional flavors of Polish cuisine.

The calculated Cramér’s V coefficient values for American, Arabic, Indian, Mexican, and Italian cuisines fall within the range of <0.2; 0.4, indicating that the strength of the association between the statistical feature “preferred world cuisine” and the statistical feature “age” is low. The Cramér’s V values for Chinese, Japanese, and Mediterranean cuisines fall within the range of <0–0.2, indicating that the strength of the association between the statistical feature “preferred world cuisine” and the statistical feature “age” is weak (Table 4).

### 3.2. Use of Food Service Establishments

**Figure 5:**
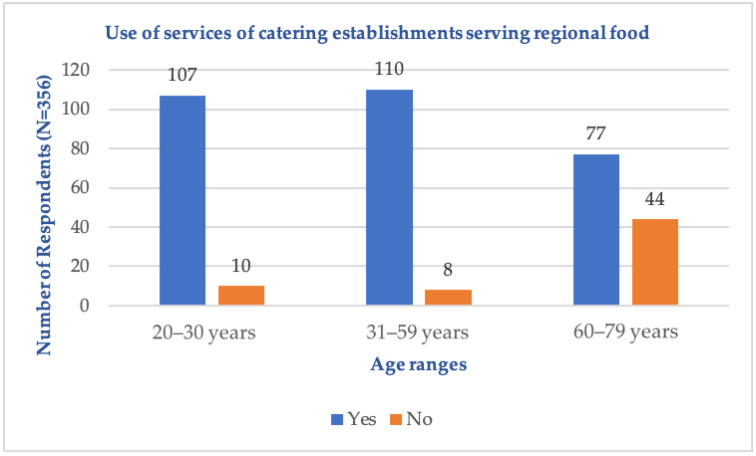
Use of services of food establishments serving regional dishes (N = 356).

In response to the question “Do you use food establishments serving regional dishes during travel?”, most respondents in all age groups answered affirmatively. In the age group 60–79, 44 respondents answered negatively. These data are presented in Figure 5. These differences may stem from varying travel motivations and culinary preferences shaped by social, economic, and technological factors.

The calculated Cramér’s V coefficient value is 0.36, indicating that the strength of the association between the statistical feature “use of services of food establishments serving regional dishes” and the statistical feature “age” in the studied general population is low. The use of services of food establishments serving regional dishes across different age groups is significantly varied (*p* < 0.01). The results are shown in Table 5.

## 4. Discussion

Poland’s accession to the European Union significantly impacted the modification of the food service market. Food establishments continuously increase their staff and modify the types of services they offer to meet the growing demands of consumers. Changes in the environment and the development of food services influence eating styles, dietary preferences, and ways of spending free time. Poles use food service establishments more frequently than they did a few decades ago [28,29].

In recent years, there has been a significant increase in the popularity of international cuisine, which is reflected in the menus of many dining establishments. Moreover, studies suggest that the rise in confidence regarding food quality and safety standards in restaurants following Poland’s accession to the European Union has had a substantial impact on consumer preferences. The growing number of restaurants offering regional and seasonal cuisine also indicates significant shifts in eating styles, emphasizing the locality and authenticity of the ingredients and products used [30,31,32].

The food environment plays an important role in shaping consumers’ dietary habits, which is why it is essential to continuously improve the operation of food establishments and the dishes they prefer [33].

Interest in world cuisines has led to an increasing number of restaurants serving dishes typical of various ethnic cuisines. Analysis of the obtained results showed that the most frequently chosen national cuisines by respondents were Polish (233 respondents), Italian (179 respondents), Mediterranean (79 respondents), American (78 respondents), and Chinese (72 respondents). These results are similar to those obtained in the “Polska na Talerzu 2018” report, where respondents identified Polish (79% of respondents), Italian (56%), Arabic and American (28%), and Chinese (20%) cuisines as the most preferred [34].

Additionally, a Euromonitor report indicates that consumers are increasingly opting for dishes offering added value, such as those with health benefits or products with so-called “clean labels”. These shifts have contributed to the growing prominence of establishments serving Mediterranean, American, and Chinese specialties in Poland, as confirmed by the findings of this study [35].

Culinary tourism participants pay particular attention to selecting places where they can try local dishes typical of the visited region. In this study, to verify whether respondents are interested in culinary traditions while traveling, they were asked “Do you use food establishments serving regional dishes during travel?” The majority responded affirmatively (83.6% of respondents). The study by Stokłosa and Krupa shows that to a similar question, 55% of respondents answered “yes”, 11% “not in every country”, 19% answered “occasionally”, and 15% gave a negative answer [36].

The analysis of respondents’ dietary preferences revealed diverse consumer choices regarding products such as ayran, tempeh, and chorizo. This study’s findings indicate that prior familiarity with a product significantly influences quality assessment, a conclusion supported by the existing literature. Subali et al. demonstrated that products like tempeh can be highly valued by consumers when their sensory properties are appropriately enhanced through seasoning or preparation modifications [37].

The overall quality of ayran was rated as good (Me = 4.2), with dietitians awarding maximum points to quality indicators such as color, aroma, and appearance. In the studies by Wichrowska and Wojdyła, yogurts with high-fat content received the highest organoleptic value (color, taste, aroma, and texture) [38].

Modern research on fermented products, such as ayran, indicates that the type of bacterial cultures used significantly affects both organoleptic quality and nutritional value. A 2023 study found that ayran prepared with mixed probiotic cultures received the highest sensory evaluations due to improved texture and increased probiotic content. These attributes make such products more appealing to consumers, who value both taste and nutritional benefits [38,39].

The overall quality of rice noodles was rated as good (Me = 4.8), with dietitians giving the quality indicator “taste” the maximum score (Me = 1.5). Other quality indicators scored close to the maximum. In the study by Tomiło et al., the quality of five different types of instant rice noodles was assessed. The quality of all tested noodles, prepared according to the method suggested by the manufacturers, was rated as good [40].

Contemporary research highlights the significant role of processing technologies and modifications in enhancing the organoleptic quality of rice noodles. Chen et al. demonstrated that germinating brown rice substantially improves sensory attributes—such as taste, consistency, and texture—by activating endogenous enzymes and increasing bioactive compounds, like γ-aminobutyric acid (GABA). This process also reduces bitterness and minimizes starch loss during cooking, thereby elevating consumer acceptance of these products [41].

Similar findings were reported by Halim et al., who emphasized the importance of additives like konjac glucomannan in enhancing the consistency and texture of rice noodles, making them more appealing to consumers seeking products with increased nutritional value [42].

These findings align with previous observations by Tomiło et al., confirming that appropriate processing and functional additives can significantly enhance the organoleptic evaluation and overall quality of rice noodles. Further research into innovative processing technologies is recommended to increase the appeal of these products among consumers [40].

The overall quality of tempeh was rated as satisfactory (Me = 3.8), with color being the highest-rated quality indicator (Me = 0.5). Significant differences in the evaluation of various quality indicators may result from the fact that students do not constitute a group with proven sensory sensitivity, and their individual taste preferences may have interfered with an objective organoleptic analysis of the products. Kuligowski and Nowak studied consumer preferences for tempeh prepared from soy and beans, and their results suggest that tempeh made from soybeans and subjected to thermal processing, such as frying, is better rated by Polish consumers [43]. Tempeh is popular worldwide due to its health benefits, taste, ease of preparation, and low cost [44]. Subali et al. demonstrated that tempeh is a potential product that can enrich the diet of athletes due to its high protein, vitamin, antioxidant, and probiotic content and valuable calcium sources [37].

The findings of Aaslyng and Højer indicate that the diverse sensory profiles of tempeh stem from the use of various raw materials and fermentation methods. Consumers showed a preference for tempeh when it was presented as a unique protein product rather than as a meat substitute in recipes [45].

Tan et al. demonstrated that combining soybeans with red kidney beans during fermentation can significantly enhance sensory attributes, including texture and taste, making the product more acceptable to consumers [46].

Additionally, Chen et al. emphasized in their research that the fermentation process in tempeh production can increase the content of γ-aminobutyric acid (GABA), making it a valuable product for individuals seeking functional foods [41].

The overall quality of chorizo was rated as good (Me = 4.6). The highest-rated quality indicators for chorizo were external appearance and texture (Me = 0.25). A similar overall quality of chorizo sausage was shown in the study by Lilic et al. [47]. In the mentioned study, the impact of reducing sodium chloride on the sensory quality of chorizo sausages was examined. Sausages with typical sodium chloride content showed the highest organoleptic quality. In sausages where sodium chloride was replaced by potassium and ammonium chloride, a bitter taste was detected, which only affected the overall quality of chorizo and not the taste, color, or aroma.

The non-parametric Mann–Whitney U test showed that individuals previously familiar with ayran rated its taste higher than those who had not tried it before. This result cannot be compared with the literature data due to the lack of similar analyses by other authors.

The final question aimed to determine how frequently respondents would purchase the tested food products. Most respondents were critical of ayran and tempeh, declaring they would not buy these products. Slightly more respondents would purchase chorizo and rice noodles, although it would not be a frequent purchase. In their study on consumer acceptance of tempeh products, Kuligowski and Nowak found a higher frequency of purchasing the product than in this study (42% of respondents would not buy tempeh, 50% would buy it regularly, and 8% several times a year). Kuligowski and Nowak suggested that tempeh would achieve higher acceptability if seasoned during culinary preparation, especially with salt; this hypothesis was not confirmed in this study, where a small amount of salt was added during sample preparation. However, it is not excluded that pre-marinating tempeh or using intense spices could positively influence the product’s rating [43].

### Study Limitations and Future Directions

Future studies could go beyond a cross-sectional approach and include longitudinal or experimental studies to capture changing preferences and investigate how time and exposure to products affect acceptance. Experimental studies with controlled trials of different product preparation methods could reveal the influence of variable exposure on taste preference formation, especially for products with a more intense profile, such as tempeh. Additionally, a demographic analysis could help determine which age or social groups are most open to new flavors and world cuisines.

However, this study has some limitations, such as the social desirability effect, where respondents might indicate preferences considered socially desirable or trendy, potentially affecting their answers. Additionally, the reliance on sensory analysis experiments and questionnaire surveys limits this study’s innovation, as it does not incorporate more diverse research methods. Future analyses should account for these potential biases by integrating experimental or longitudinal research approaches. Such approaches could more accurately examine how consumer preferences evolve under the influence of factors such as culinary experience, migration, or the increasing availability of foreign products. Expanding the methodological framework would contribute to a deeper understanding of consumption dynamics, offering more comprehensive insights into the reasons behind changing preferences and enhancing this study’s overall impact.

## 5. Conclusions

The consumer assessment of the attractiveness of selected sensory characteristics, conducted using a five-point method, showed that the highest-rated food product by respondents was rice noodles, while tempeh received the lowest scores. The vast majority of respondents indicated that they would not purchase tempeh or ayran. The most preferred world cuisines among consumers in the 23–30 and 31–59 age groups were Italian and Polish cuisine, whereas respondents in the 60–79 age group preferred Polish cuisine. Respondents most frequently cited travel and dining in food establishments as the most common ways to explore the cuisines of other nations.

This study revealed Polish consumers’ preferences for selected world cuisine products and their willingness to explore new flavors, which has significant implications for the public health and consumer protection sectors. The results indicate the high popularity of Italian and Polish cuisines, particularly among younger and middle-aged individuals, while older respondents tend to prefer Polish cuisine. Such preferences may help better understand the growing importance of culinary tourism, which not only satisfies taste needs but also supports the development of intercultural relationships and enriches consumers’ culinary identity.

This study’s findings can serve as practical guidance for the food and gastronomy industries in Poland. The particularly high rating of rice noodles suggests that Asian cuisine products could find a broad audience. Food companies may consider introducing more similar products, emphasizing their health benefits and ease of preparation. Conversely, the low acceptance level of tempeh and ayran indicates a need for consumer education on the health benefits of these products and potential flavor adjustments for Polish consumers to increase their appeal.

This study makes a significant contribution to the analysis of culinary preferences in Poland by including data on sensory preferences. Further research should examine the impact of health education on the acceptance of lesser-known products and explore how global health trends (e.g., the popularity of plant-based diets) influence interest in world cuisine products.

The findings of this study provide valuable implications for the tourism industry in Poland, particularly in the development of culinary tourism. The strong preference for Italian and Polish cuisines among Polish consumers highlights the opportunity for targeted marketing strategies and the promotion of these cuisines to enhance tourist experiences. Additionally, the emphasis on traditional Polish cuisine underscores its potential as a cornerstone for cultural tourism, offering visitors authentic regional dining experiences. Insights into consumer familiarity and sensory preferences for products, like rice noodles and tempeh, suggest opportunities for both product adaptation and consumer education, aligning with global trends in sustainability and plant-based diets. By integrating these findings, the tourism industry in Poland can diversify its offerings, attract a broader audience, and position itself as a destination that combines rich cultural heritage with innovative culinary experiences.

## Figures and Tables

**Table 1:** Preparation of products for sensory evaluation and sample size.

Product	Preparation and Sample Size
Ayran	Chilled beverage. Sample size of 30 mL.
Rice noodles	Soaked in boiling water for 5 min, drained, rinsed with cold water. Sample size of 30 g.
Chorizo	Sliced into 1 mm thick slices.
Tempeh	Fried for 5 min on each side in a small amount of oil, seasoned with salt. Sample size of 14 g.

**Table 2:** Overall quality of the tested products.

Product	Quality Indicator	IC	N	$\bar{x}\;\pm \;SD$	Me (R_k_)	$x_{min}÷$ x_max_
Ayran	Color	0.1	51	0.47 ± 0.04	0.5 (0.1)	0.4–0.5
	Aroma	0.15	51	0.66 ± 0.1	0.75 (0.15)	0.45–0.75
	Texture	0.25	51	0.95 ± 0.25	1.0 (0)	0.25–1.25
	Appearance	0.15	51	0.66 ± 0.13	0.75 (0.15)	0.3–0.75
	Taste	0.35	51	1.32 ± 0.28	1.4 (0.35)	0.7–1.5
	Total Points	1	51	4.07 ± 0.5	4.15 (0.65)	2.35–5
Rice noodles	Color	0.1	51	0.47 ± 0.05	0.5 (0.1)	0.4–0.5
	Aroma	0.1	51	0.47 ± 0.6	0.5 (0.1)	0.3–0.5
	Texture	0.3	51	1.28 ± 0.28	1.5 (0.6)	0.9–1.5
	Appearance	0.2	51	0.95 ± 0.1	1 (0.2)	0.6–1
	Taste	0.3	51	1.41 ± 0.23	1.5 (0)	0.3–1.5
	Total Points	1	51	4.57 ± 0.49	4.8 (0.8)	3.1–5
Tempeh	Color	0.1	51	0.43 ± 0.01	0.5 (0.1)	0.2–0.5
	Aroma	0.1	51	0.38 ± 0.06	0.4 (0.1)	0.3–0.5
	Texture	0.3	51	1.11 ± 0.28	1.2 (0.3)	0.6–1.5
	Appearance	0.2	51	0.7 ± 0.19	0.8 (0.2)	0.4–1
	Taste	0.3	51	1.16 ± 0.31	1.2 (0.3)	0.3–1.5
	Total Points	1	51	3.79 ± 0.6	3.8 (0.8)	2.4–4.9
Chorizo	External Appearance	0.05	51	0.22 ± 0.03	0.25 (0.05)	0.15–0.25
	External Structure	0.15	51	0.65 ± 0.1	0.6 (0.15)	0.45–0.75
	Color	0.15	51	0.66 ± 0.1	0.75 (0.15)	0.45–0.75
	Texture	0.05	51	0.22 ± 0.03	0.25 (0.05)	0.15–0.25
	Aroma	0.2	51	0.86 ± 0.18	1 (0.2)	0.4–1
	Taste	0.4	51	1.7 ± 0.38	2 (0.4)	0.8–2
	Total Points	1	51	4.33 ± 0.62	4.55 (1)	2.65–5

Legend: IC—Importance Coefficient; $\bar{x}$—symbol for the arithmetic mean; SD—standard deviation; [n]—sample size; Me—symbol for the media.

**Table 3:** Quality vs. prior familiarity with the product.

Product	Quality	No			Yes			*p*-Value
		N	$\bar{x}$ ± SD	Me (R_k_)	N	$\bar{x}$ ± SD	Me (R_k_)	
Ayran	Color	44	0.47 ± 0.04	0.5 (0.1)	7	0.49 ± 0	0.5 (0)	0.59
	Aroma	44	0.66 ± 0.04	0.7 (0.15)	7	0.66 ± 0.15	0.75 (0.15)	0.91
	Texture	44	0.96 ± 0.25	1.0 (0)	7	0.86 ± 0.5	1.0 (0.5)	0.35
	Appearance	44	0.65 ± 0.14	0.8 (0.15)	7	0.69 ± 0.15	0.75 (0.15)	0.82
	Taste	44	1.28 ± 0.26	1.4 (0.35)	7	1.6 ± 0.35	1.75 (0.35)	0.01
Rice noodles	Color	3	0.47 ± 0.06	0.5 (0.1)	48	0.47 ± 0.05	0.5 (0.1)	0.97
	Aroma	3	0.43 ± 0.06	0.4 (0.1)	48	0.47 ± 0.06	0.5 (0.05)	0.29
	Texture	3	1.1 ± 0.35	0.9 (0.6)	48	1.29 ± 0.27	1.5 (0.6)	0.34
	Appearance	3	1.0 ± 0	1.0 (0)	48	0.94 ± 0.1	1.0 (0.2)	0.45
	Taste	3	0.9 ± 0.6	0.9 (1.2)	48	1.44 ± 0.15	1.5 (0)	0.09
Tempeh	Color	47	0.42 ± 0.1	0.5 (0.2)	4	0.45 ± 0.06	0.45 (0.1)	0.82
	Aroma	47	0.38 ± 0.06	0.4 (0.1)	4	0.38 ± 0.05	0.4 (0.05)	0.99
	Texture	47	1.12 ± 0.28	1.2 (0.6)	4	1.05 ± 0.17	1.05 (0.3)	0.66
	Appearance	47	0.72 ± 0.19	0.8 (0.2)	4	0.6 ± 0.16	0.6 (0.2)	0.24
	Taste	47	1.15 ± 0.31	1.2 (0.6)	4	1.35 ± 0.17	1.35 (0.3)	0.24
Chorizo	External Appearance	19	0.22 ± 0.02	0.2 (0.05)	32	0.23 ± 0.03	0.25 (0.05)	0.28
	External Structure	19	0.62 ± 0.11	0.6 (0.15)	32	0.67 ± 0.1	0.75 (0.15)	0.11
	Color	19	0.63 ± 0.11	0.6 (0.15)	32	0.68 ± 0.09	0.75 (0.15)	0.14
	Texture	19	0.23 ± 0.03	0.25 (0.05)	32	0.22 ± 0.04	0.25 (0.05)	0.28
	Aroma	19	0.81 ± 0.19	0.8 (0.4)	32	0.89 ± 0.18	1 (0.2)	0.24
	Taste	19	1.64 ± 0.4	1.6 (0.8)	32	1.75 ± 0.36	2 (0.4)	0.36

**Table 4:** Analysis of the relationship of the type of preferred world cuisine by age.

World Cuisine	Number of People (%)			*p*-Value	V Cramer	Strenght of Relationship
	23–30 years	31–59 years	60–79 years			
American	32.5	28.8	5	*p* < 0.01	0.3	Low
Arabic	10.2	25.4	3.3	*p* < 0.01	0.28	Low
Chinese	23.1	27.1	10.7	*p* < 0.01	0.17	Weak
Indian	4.3	13.6	1.7	*p* < 0.001	0.21	Low
Japanese	12	11	2.5	*p* = 0.01	0.15	Weak
Mexican	14.5	20.3	0.8	*p* < 0.01	0.25	Low
Polish	65.8	65.3	65.3	*p* = 0.99	-	-
Mediterranean	26.5	28.8	11.6	*p* < 0.01	0.18	Weak
Italian	67.5	61	23.1	*p* < 0.01	0.39	Low

**Table 5:** Analysis of the relationship between the use of services of food establishments serving regional dishes by age.

Use of Services of Food Establishments Serving Regional Dishes	Number of People (%)			*p*-Value	V Cramer	Strength of Relationship
	23–30 years	31–59 years	60–79 years			
Yes	91.5	93.2	63.6	*p* < 0.01	0.36	Low
No	8.5	8.6	36.4			

## Data Availability

The original contributions presented in this study are included in the article. Further inquiries can be directed to the corresponding author.
